# The impact of the COVID-19 pandemic on patient complaints within one Irish teaching hospital

**DOI:** 10.1007/s11845-023-03282-0

**Published:** 2023-02-14

**Authors:** Emily O’Dowd, Sinéad Lydon, Marie E. Ward, Maria Kane, Una Geary, Chris Rudland, Paul O’Connor

**Affiliations:** 1 Discipline of General Practice, School of Medicine, National University of Ireland Galway, Galway, Ireland; 2 Irish Centre for Applied Patient Safety and Simulation, School of Medicine, National University of Ireland Galway, Galway, Ireland; 3 School of Medicine, National University of Ireland Galway, Galway, Ireland; 4 Quality and Safety Improvement Directorate, St James’s Hospital, Dublin, Ireland; 5 University of Dublin, Dublin, Ireland; 6 National Complaints Governance and Learning Team, Health Service Executive, Catherine Street, Limerick, Ireland; 7 Department of Surgical Affairs, RCSI, Dublin, Ireland

**Keywords:** Complaints, Healthcare quality, Hospital care, Patient insights, Patient safety

## Abstract

**Background:**

The coronavirus disease 2019 (COVID-19) pandemic dramatically impacted the delivery of hospital care in terms of quality and safety.

**Objectives:**

To examine complaints from two time points, quarter 4 (Q4) 2019 (pre-pandemic) and Q4 2020 (second wave), and explore whether there was a difference in the frequency and/or content of complaints.

**Methods:**

A retrospective analysis of complaints from one Irish hospital was conducted using the Healthcare Complaints Analysis Tool (HCAT). Within each complaint, the content, severity, harm reported by the patient, and stage of care were categorised. The complaints were analysed using descriptive statistics and chi-square tests of independence.

**Results:**

There were 146 complaints received in Q4 2019 and 114 in Q4 2020. Complaint severity was significantly higher in Q4 2019 as compared to Q4 2020. However, there were no other significant differences. Institutional processes (e.g. staffing, resources) were the most common reason for complaints (30% in Q4 2019 and 36% in Q4 2020). The majority of complaints were concerned with care on the ward (23% in Q4 2019 and 31% in Q4 2020).

**Conclusions:**

The severity of complaints was significantly higher in Q4 2019 than in Q4 2020, which requires further exploration as the reasons for this are unclear. The lack of a difference in the frequency and content of complaints during the two time periods was unexpected. However, this may be linked to a number of factors, including public support for the healthcare system, existing system-level issues in the hospital, or indeed increased staff collaboration in the context of the COVID-19 crisis.

## Introduction

With the onset of the coronavirus disease 2019 (COVID-19) pandemic, the delivery of patient care was dramatically altered both in Ireland and internationally [1, 2]. Efforts to reduce the spread of the virus, such as lockdowns, the migration to virtual healthcare consultations, the cancellation of elective care, and visitor restrictions, all impacted normal healthcare delivery [1, 3]. Healthcare staff experienced immense pressure during the early stages of the pandemic, with limited initial knowledge about the features of the virus or its transmission, a shortage of appropriate personal protective equipment (PPE), and the psychological strain of treating patients who were very sick with COVID-19 [4–7]. Two years on from the beginning of the pandemic, the true extent of the impact the virus has had on the safety and quality of care provided to patients is beginning to be understood [8, 9].

Patient safety was perceived by staff in some studies to have been negatively affected by the pandemic as a result of changes such as new infection prevention control measures and the redeployment of staff to areas in which they had limited clinical expertise [9, 10]. During the pandemic, efforts were made to continue the monitoring of patient safety and quality of care using staff surveys, incident reporting, and patient feedback forms [10–12]. However, the high workload during the initial and subsequent waves of the pandemic meant that often, quality and safety improvement efforts were not carried out, and some patient safety events increased, possibly due to a lack of direct observation by staff and patient advocates [13–15]. This lack of focus on quality and safety improvement during the pandemic is very understandable. However, throughout the pandemic, patient complaints (written expressions of dissatisfaction with the care an individual or their family member has received) were still submitted and responded to, and they may be a useful source of quality and safety information [16].

While traditionally, many measures of quality and safety have been staff-reported, there has been an increased focus on including the patient perspective to improve care [17, 18]. This may be particularly important during a time of immense strain on the healthcare system such as a pandemic [13]. Complaints have typically been seen as a public relations issue and are resolved on a case-by-case basis [19]. They do, however, have the potential to be useful as a measure of quality and safety at the hospital, or healthcare system, level [16, 19, 20]. To this end, the Healthcare Complaints Analysis Tool (HCAT) was developed to provide a structured approach to analysing complaints in order to identify trends and highlight points in care that require immediate attention [21]. In Ireland, the value of using complaints as a source of safety information is evident, with the Health Service Executive (HSE) receiving over 15,000 complaints relating to hospital care in 2019 [22].

Complaints have been analysed in both hospital [23–25] and primary care [26] settings in Ireland prior to the COVID-19 pandemic. However, it is not known if, and how, the COVID-19 pandemic may have impacted complaints. Therefore, our study reports the analysis of patient complaints received by a single hospital in Ireland at two time points—quarter 4 (Q4) of 2019, prior to the onset of the COVID-19 pandemic, and Q4 of 2020, during the second wave of COVID-19 in Ireland. The aim of this study was to examine and explore complaints pre- and during the COVID-19 pandemic in terms of their frequency and severity and to analyse the content of complaints across these two time points.

## Method

### Design

This is a retrospective database analysis of complaints received by one large teaching hospital in Ireland in Q4 2019 and Q4 2020.

### Context

This study was conducted using hospital complaints from Q4 2019 to Q4 2020. Hospital care during each of these two time periods was strikingly different and requires some contextualisation. In Q4 2019 (prior to the pandemic), care as usual was delivered in Irish hospitals. There are long-standing issues with bed capacity in the Irish healthcare system, particularly in the winter season. Therefore, the system was under the typical strain for the time of year, with higher admissions than in the previous years [27]. The COVID-19 pandemic started in Ireland in March 2020 [27]. Elective healthcare was postponed, with staff redeployed to manage the virus and its impacts [8]. Visitors to the hospitals were also restricted, and patients were mostly unaccompanied by family members or advocates [27]. During Q4 2020, Ireland entered a second pandemic wave following a summer of relatively few restrictions. The country was placed back on strict lockdown in October 2020 [28]. Although elective care continued, there was a large backlog of patients as a result of the cancellations from the first wave [29]. Admissions to hospitals across Ireland were lower in quarters 2 to 4 of 2020 as compared to admissions immediately before the onset of the COVID-19 pandemic, as people tended to avoid attending emergency departments (EDs) except for urgent emergencies [29]. Attendance and admission from EDs nationally experienced a 19% reduction (81,712 fewer presentations than in quarter 1 of 2020). In the hospital from which we analysed complaint data, ED attendance, inpatient admissions, and outpatient clinic attendance fell from 117,379 in Q4 2019 to 107,247 in Q4 2020 (a 9% reduction). ED attendances alone in this hospital reduced by 1597 (− 13%) from Q4 2019 to Q4 2020.

### Sample

All of the complaints received by a large Irish teaching hospital in Q4 2019 and Q4 2020 were collated for analysis.

### Ethics

This study was approved by the NUIG Research Ethics Committee (REC) and the Hospital Research and Innovation Office (R&I ref: 7071).

### Procedure

Complaints from Q4 2019 and Q4 2020 were extracted from the hospital’s complaint database by staff from the hospital’s Quality and Safety Improvement Directorate (QSID). Initially, a sample of these complaints (10%) was redacted and sent to the National University of Ireland, Galway (NUIG) research team for double-coding to ensure standardisation across coders. Following the double-coding of the complaints, which resulted in 86% agreement, the remaining complaints were coded by MEW and MK (each coding half of the remaining complaints). The coded data were then sent to EOD for statistical analysis. All of the authors have been trained to use the HCAT [30]. One author (EOD) has considerable experience coding complaints using the HCAT from a previous study in which 641 complaints were analysed [25].

### Complaint coding

The coding of the complaints was carried out using HCAT. The process followed the steps outlined in the HCAT user guide written by the developers of the tool [21]. Firstly, the HCAT is used to identify the distinct domain(s) (clinical, management, and relationship) of the unique issues within healthcare complaints. Next, these domains are broken down into categories (quality, safety, environment, institutional processes, listening, communication, and respect and patient rights). There can be more than one issue identified in a single complaint. Therefore, it may be necessary to use more than one category or domain in order to classify all of the issues within a complaint. The severity of the issue(s) identified within the complaint is then classified from low (1) to high (3). The stage, or stages of care, at which the issue occurred is identified (admissions, examination and diagnosis, care on the ward, operation or procedures, discharge, or ‘other’). Finally, the overall harm to the patient, as reported by the complainant, is determined. Unlike the other aspects of the HCAT, this is measured at a complaint level, with only one rating per complaint. An assessment of harm is made from minor (1: minimal intervention or treatment required) to catastrophic (5; death or multiple/permanent injuries). A classification of 0 is given if no harm was reported.

### Analysis

All coded data were inputted into the R statistical software for analysis [31]. A descriptive analysis of the complaints was completed. This analysis identified the frequency of the categories, stages of care, severity, and harm reported in complaints. The individuals that made the complaint (i.e. patient, family member, other advocates, etc.) were also analysed, as were the staff members who were involved in the complaint. Chi-square tests were conducted to determine if there were trend differences between Q4 2019 and Q4 2020 complaints for the category of complaint, stages of care, harm, and severity.

## Results

### Descriptive statistics

Descriptive statistics of the complaint data from Q4 2019 to Q4 2020 are shown in Table 1. There were a total of 146 complaints received by the hospital in Q4 2019 and 114 in Q4 2020—a total of 260 complaints. The majority of complaints in both Q4 2019 and Q4 2020 were made by patients themselves, followed by adult children of patients. A small number of complaints each year were made by patient advocates other than immediate relatives. Complaints were most frequently directed towards clinical staff with whom patients engage on a daily basis while in the hospital, i.e. doctors (41%) and nurses (29%).

**Table 1:** Descriptive statistics of complaints

	***N* complaints (%)**		
	**2019**	**2020**	**Total**
**Complainant**			
Patient	93 (64%)	65 (59%)	158 (62%)
Spouse/partner of patient	7 (5%)	7 (6%)	14 (5%)
Parent of patient	5 (3%)	7 (6%)	12 (5%)
Child of patient	29 (20%)	27 (24%)	56 (22%)
Other family members	10 (7%)	2 (2%)	12 (5%)
Other advocates	1 (1%)	3 (3%)	4 (1%)
**Staff category**			
Doctors	28 (39%)	21 (42%)	49 (41%)
Nurses	23 (32%)	12 (24%)	35 (29%)
Admin/clerical staff	10 (14%)	9 (18%)	19 (16%)
Security	2 (3%)	5 (10%)	7 (6%)
Other clinical staff^a^	5 (7%)	1 (2%)	6 (5%)
Other/unclear	3 (4%)	1 (2%)	4 (3%)
**Harm**			
0—no harm reported	66 (45%)	56 (49%)	122 (47%)
1—minimal harm	15 (10%)	10 (9%)	25 (10%)
2—minor harm	33(23%)	31 (27%)	64 (25%)
3—moderate harm	19 (13%)	9 (8%)	28 (11%)
4—major harm	8 (5%)	4 (4%)	12 (5%)
5—catastrophic harm	5 (3%)	2 (2%)	7 (3%)
No information	0 (0%)	2 (2%)	2 (1%)

^a^For example, healthcare assistant, phlebotomist, and pharmacist

These complaints often contained more than one unique issue, with a total of 457 issues across the complaints (mean = 1.76, SD = 1.04). There were a total of 272 issues across the complaints in Q4 2019 (mean = 1.86, SD = 1.11) and 185 in Q4 2020 (mean = 1.62, SD = 0.93). Descriptive statistics on the HCAT characteristics analysed at an issue level are found in Table 2.

**Table 2:** HCAT characteristics of issues within complaints

	***N issues* (%)**		
	**2019**	**2020**	**Total**
**Category**			
Quality	35 (13%)	20 (11%)	55 (12%)
Safety	37 (14%)	15 (8%)	52 (11%)
Environment	18 (7%)	14 (8%)	32 (7%)
Institutional processes	81 (30%)	66 (36%)	147 (32%)
Communication	41 (15%)	27 (15%)	68 (15%)
Listening	19 (7%)	15 (8%)	34 (7%)
Respect and patient rights	41 (15%)	28 (15%)	69 (15%)
**Severity**			
Low	64 (24%)	75 (40%)	139 (30%)
Medium	146 (54%)	84 (45%)	230 (50%)
High	59 (22%)	27 (14%)	86 (19%)
Missing data	0 (0%)	2 (1%)	2 (1%)
**Stage**			
1. Admission	75 (28%)	37 (20%)	112 (25%)
2. Examination/diagnosis	57 (21%)	34 (18%)	91 (20%)
3. Care on the ward	63 (23%)	57 (31%)	120 (26%)
4. Operation/procedures	28 (10%)	17 (9%)	45 (10%)
5. Discharge	12 (4%)	8 (4%)	20 (4%)
6. Unknown or other	30 (11%)	31 (17%)	61 (13%)
Multiple stages	4 (1%)	1 (1%)	5 (1%)
Missing data	3 (1%)	0 (0%)	3 (1%)

### Category of complaint

#### Descriptive analysis

All seven HCAT categories were represented across both time points and were typically consistent across the 2 years. In both Q4 2019 and Q4 2020, institutional process issues were the most prevalent within the complaints, representing 30% and 36% of issues, respectively. Issues coded as ‘Institutional Processes’ frequently encompassed long waiting times in the emergency department, problems with healthcare fees, and lengthy waiting lists for specialist care. Further detail on these can be seen in Table 3. One patient requested information on ‘why [the patient’s] clinic appointment was cancelled and when [they] will be seen’ (*rated as severity 1—low*)*.* Communication and respect and patient rights were the next two most commonly reported categories of issues across the two time points (15% of issues each year). Communication issues included the patient receiving ‘no further correspondence’ (severity level 1) from the hospital about a rescheduled appointment. Respect and patient rights issues included a patient reporting their perception that a staff member’s attitude was ‘dismissive and very rude throughout’ the interaction (rated as severity 2—medium).

**Table 3:** Categories and examples from the HCAT guide

**HCAT category**	**Definition**	**High severity example from the HCAT guide**
Quality	Clinical standards of healthcare staff behaviour	Patient left in own waste in bed
Safety	Errors, incidents, and staff competencies	Failure to coordinate time-critical decision
Environment	Problems in the facilities, services, clinical equipment, and staffing levels	Patient relocated due to bed shortage
Institutional processes	Problems in bureaucracy, waiting times, and accessing care	Emergency phone calls not responded to
Communication	Absent or incorrect communication from healthcare staff to patients	Patient given wrong test results
Listening	Healthcare staff disregard or do not acknowledge information from patients	Critical patient-provided information repeatedly dismissed
Respect and patient rights	Disrespect or violations of patient rights by staff	Private information shared with members of the public

#### Inferential analysis

While the initial descriptive analysis gave some insight into the differences and similarities in complaint trends between Q4 2019 and Q4 2020, inferential statistics were also required to determine if there were any statistically significant differences. A series of chi-square tests of independence were run in order to explore these, the results of which are presented in Table 4. There were no significant differences found between Q4 2019 and Q4 2020 on the HCAT categories of the complaints.

**Table 4:** Chi-square tests of differences between 2019 and 2020

**HCAT aspect**	***χ***^**2**^	***df***	***p* value**
Harm	0.45	1	.5
Categories	4.84	6	.56
Stage of care	9.08	6	.17
Severity	15.24	2	.0005*

^*^Significant at *p* < .05

### Severity

#### Descriptive analysis

The complaints from the two time points contained issues with severity levels ranging from low (1) to high (3). In both years, issues were predominantly categorised as being ‘medium severity’, with 54% of issues in Q4 2019 at this severity level and 45% in Q4 2020. Medium-severity issues included patients reporting that they felt their questions were ‘dismissed as irrelevant’ by clinicians and ‘being told after waiting for hours that I wouldn’t be seen that day’. Interestingly, there was a higher proportion of ‘Low severity’ issues in 2020 (40%) compared to 2019 (24%). A breakdown of the severity of issues by category is found in Table 5.

**Table 5:** Breakdown of issue categories by severity

**Categories**	***N* issues (%)**			
	**Low severity**	**Medium severity**	**High severity**	**No data**
**2019**				
Quality	7 (20%)	16 (46%)	12 (34%)	0 (0%)
Safety	4 (11%)	22 (59%)	11 (30%)	0 (0%)
Environment	6 (33%)	11 (61%)	1 (6%)	0 (0%)
Institutional processes	18 (22%)	41 (51%)	22 (27%)	0 (0%)
Communication	12 (29%)	26 (63%)	3 (7%)	0 (0%)
Listening	4 (21%)	11 (58%)	4 (21%)	0 (0%)
Respect and patient rights	15 (37%)	21 (51%)	5 (12%)	0 (0%)
**2020**				
Quality	5 (25%)	8 (40%)	7 (35%)	0 (0%)
Safety	4 (27%)	5 (33%)	6 (40%)	0 (0%)
Environment	6 (43%)	8 (57%)	0 (0%)	0 (0%)
Institutional processes	37 (56%)	21 (32%)	7 (10%)	1 (2%)
Communication	13 (48%)	13 (48%)	1 (4%)	0 (0%)
Listening	4 (26%)	9 (60%)	1 (7%)	1 (7%)
Respect and patient rights	10 (36%)	14 (50%)	4 (14%)	0 (0%)

#### Inferential analysis

**Fig. 1:**
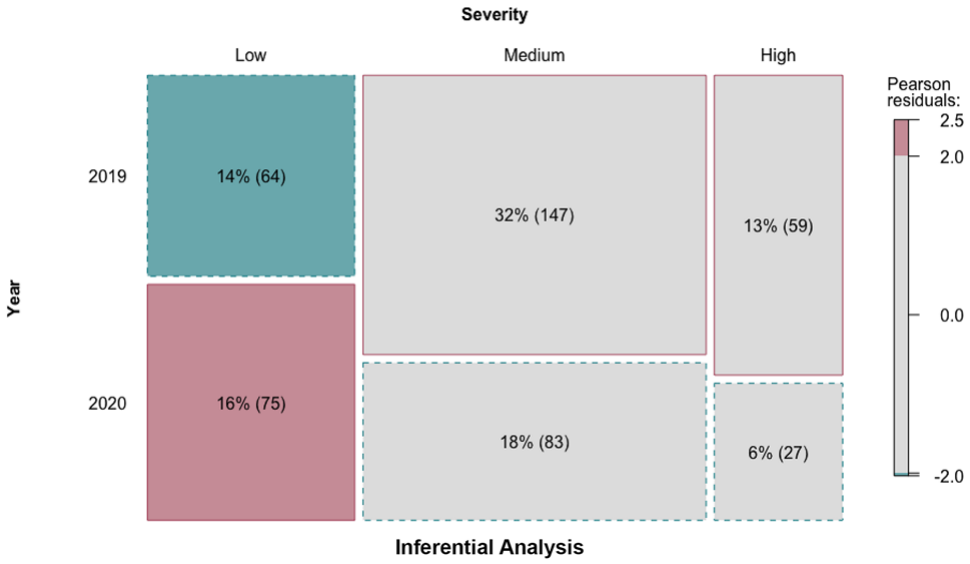
Demonstrating difference in severity of complaints between 2019 and 2020

As can be seen in Table 4, only the severity of issues differed significantly between Q4 2019 and Q4 2020. This is explored further in Fig. 1. To interpret Fig. 1, the solid line around a box indicates that the number of complaints is higher than statistically expected, while the dotted line indicates that the number is fewer than expected. The colour of the box indicates the size of the difference between observed and expected (i.e. the residuals), with the blue box being much lower than expected and the red box being much higher than expected. The mosaic plot in Fig. 1 highlights that based on observed versus expected numbers of issues in the 2 years, there were more than expected low-severity complaint issues observed in Q4 2020 and more than expected medium- and high-severity complaint issues observed in Q4 2019. In turn, there were also fewer than expected medium- and high-severity complaint issues in Q4 2020. Therefore, there was an overall decrease in the severity of complaints in Q4 2020 compared to Q4 2019.

### Stages of care

#### Descriptive analysis

The HCAT stages of care encompass all steps of the patient journey within secondary care. Each of the stages was represented in both Q4 2019 and Q4 2020 and in similar proportions. There were some differences, with ‘Admissions’ representing the most commonly complained about the stage of care in Q4 2019 and ‘Care on the ward’ in Q4 2020. Admission issues frequently related to waiting for treatment in the emergency department (‘I have already been here for over 1.5 h and no one has seen me’ [severity 2]), and care on the ward issues often related more directly to clinical care (e.g. ‘my mother fell while going to the toilet’ [severity 3]) or issues with the environment (‘I was left waiting on a trolley in the corridor’ [severity 3]).

#### Inferential analysis

No significant difference was identified between complaint stages of care in Q4 2019 and Q4 2020 (see Table 4).

### Harm

#### Descriptive analysis

Harm is categorised using the HCAT based on the patient’s perspectives, not clinical judgement. Patient-reported harm in Q4 2019 and Q4 2020 was distributed across the range in a relatively similar manner. Almost half of the complaints at each time point reported no harm (45% in 2019 and 49% in 2020). Minor harm (harm level 2 on the HCAT) was the next most commonly reported in both years (23% in 2019 and 29% in 2020) and often reflected emotional distress and upset on the part of the patient or complainant. For example, one patient wrote that they felt ‘very emotional and hurt’ (severity 2) following a negative interaction with a healthcare professional.

#### Inferential analysis

No significant difference in the harm reported by patients was identified between Q4 2019 and Q4 2020.

## Discussion

The COVID-19 pandemic has had a large impact on the delivery of patient care. However, the impact on quality and patient safety is only beginning to be understood and is hampered by the fact that often such data was not collected due to the redeployment of staff and the need to focus on the delivery of care [9]. Our study reported the analysis of complaints about a single hospital at two time points, one pre-pandemic and one during the second wave of the COVID-19 pandemic. Interestingly, there were few differences between the number of complaints per year, the issues about which patients were complaining, or the harm reported by patients in their complaints. However, there were differences in the severity of the complaints, with more than expected low-severity issues reported during the pandemic as compared to before the pandemic.

The similarities between the findings from the analysis of the complaints at the two time points were unexpected, as research suggests that patient care may have been of a lower standard than normal during the initial stages of the COVID-19 pandemic [9]. Many staff-reported measures indicated that patient safety had reduced during the pandemic due to a multitude of factors, including a lack of resources, the cancellation of many services, and staff burnout and stress [9, 32, 33]. However, some studies suggested that patient safety actually improved during the pandemic due to increased collaboration and a sense of togetherness amongst healthcare teams [34]. This increased collaboration could possibly account for the lack of differences between the two time points as staff worked to reduce the negative impact of the pandemic on patients. Additionally, by Q4 of 2020, the Irish healthcare system had been managing COVID-19 for 6 months, and the initial lack of PPE and knowledge about the virus had somewhat abated, along with the return of elective and routine care [8, 29]. Further inquiry into the standard of patient care during the pandemic is required to clarify whether the similarities in complaints across the two time points reflect a continuing level of care throughout the pandemic or whether other factors were at play that mediated this relationship. These could have included factors such as public support and understanding of a system under pressure or patients being too sick to complain. Determining whether these findings were unique to the hospital in our study would offer further system insights.

Interestingly, the two time points contained a high proportion of institutional process complaints, with this being the most common HCAT category used to classify the complaints in both Q4 2019 and Q4 2020. Despite the large systemic changes that occurred as a result of the pandemic (1), patients continued to complain about bureaucratic issues and waiting times in similar proportions, indicating that these problems did not disappear despite the reduced numbers attending hospitals during the COVID-19 pandemic [7]. A recent national study of Irish hospitals also found that institutional process issues were the predominant HCAT category in complaints from Q4 of 2019 [25]. It is evident, therefore, that these institutional process problems are long-standing systemic issues that require significant consideration to resolve at a national level and are not limited to the hospital analysed in this study. The Irish healthcare system, along with healthcare systems internationally, has been under immense pressure and facing capacity limitations since prior to the COVID-19 pandemic [35]; therefore, the resourcing of hospitals to reduce institutional process issues must be considered further in light of these findings.

While the number of admissions to hospitals in Ireland fell in 2020 compared to 2019 and previous years, it is interesting to note that, in the case of this study, the rate of complaints per admission was further reduced. Reports by the HSE have highlighted that winter 2020 had fewer admissions to hospitals than previous years, likely due to individuals’ reticence to present to the hospital out of fear of COVID-19 and also due to the reintroduction of restrictions on individual’s movements and behaviours to limit the spread of the virus [29]. It is possible that the restrictions on visitations (patients were asked to attend the hospital alone, and only limited visits were permitted), along with the national public sentiment of supporting the healthcare system, contributed to the fall in complaints. It is well established that most experiences of poor care go unreported [36]; therefore, the findings of this study (fewer complaints despite some research indicating that patient safety was negatively impacted) could indicate that patient dissatisfaction was not being expressed in the form of complaints. It is also possible that patients who attended the hospital during this time were sicker than in Q4 of 2019 and therefore less likely to complain, as people refrained from attending the hospital out of fear of COVID-19, except when considered an absolute emergency [29]. Another possible explanation would be the efforts made by staff to continue to provide high-quality, safe care to patients despite the pandemic, with reports highlighting increased collaboration within clinical teams during the pandemic [9]. Other measures of quality of care that solicit patient feedback, including sentiment analyses, have found that patients were typically less satisfied with their care in hospitals during the COVID-19 years [12]. The National Patient Experience Survey [37], conducted annually in Ireland, gathers patient-reported data on their experience of care in Irish hospitals. The 2019 survey results for this particular hospital indicated an overall rating of 8.2, rising to 8.3 in 2021, and reducing back to 8.2 in 2022. The survey did not take place in 2020. The relatively consistent and overall high rating seems to reflect the findings of this study, and examining both satisfaction through this survey and dissatisfaction through complaints could provide hospital management with a more complete view of the patient experience. This demonstrates the importance of considering a number of measures of patient safety and quality of care rather than focusing on only a single source of safety data such as complaints. Future research should delve more into patient satisfaction data as well as explore whether there has since been an increase in complaints relating to care during different phases of the COVID-19 pandemic, as public sentiment has changed and more patients feel the impact of the restrictions and cancellations within regular care.

The analysis of the severity of complaints in our study also produced the surprising finding that there were more low-severity issues than expected during Q4 2020. This needs to be further examined and contextualised. It is unclear why there were fewer medium- or high-severity complaint issues in Q4 2020 than in Q4 2019, but the low-severity issues increased. This finding was again contrary to what would have been expected due to the dramatic changes in care that came about as a result of the pandemic [1, 9, 38]. It would be expected that there would be more high-severity issues during the pandemic compared to prior to COVID-19. One possibility is that patients who complained were prompted by more minor issues to complain than in the past and were in fact being pushed up the ‘pyramid’ of dissatisfaction more quickly due to the poor care they received [36]. This does not however explain why there were fewer medium- and high-severity complaints. Factors such as the reduced attendance in hospitals, patients who did attend likely being more sick and unaccompanied by advocates, and additional efforts by staff to compensate for the disruption in care may each have contributed to this unexpected pattern. In future research, qualitative exploration of these complaints, and indeed complaints from Q4 2021 and beyond, could help in clarifying this unanticipated trend. Examining complaints that were made at later time points in the pandemic would be a useful exercise for researchers and, indeed, for individuals working on quality and patient safety in hospitals more broadly.

### Strengths and limitations

There are several strengths to this study. Firstly, the HCAT is a valid and reliable tool which has been applied successfully to many different healthcare settings [23, 25, 39, 40]. The agreement between raters in this study indicated that the HCAT was a suitable tool for the analysis of complaints made about this Irish hospital. The comparison of two time points (prior to COVID-19 and during the second Irish wave of COVID-19 infections) is another strength of this study. COVID-19 placed a massive strain on healthcare services across the world [11], and it is important to assess the impact of COVID-19 using a variety of methods [12]. The HCAT enabled a comparison between these two time points from the perspective of patients, a valuable insight when staff and the system were under such pressure. Utilising the HCAT for coding complaints is a valuable tool in understanding issues in healthcare settings. Finally, while there were very few differences between the two time points, this study has highlighted that some issues in the hospital were consistent despite the huge systemic changes brought about by the pandemic, which in turn indicates that these issues must be resolved in order to prepare for any future challenges faced by the healthcare system.

Although the use of two time points was a strength of the study, in another respect, it was also a limitation. The study may have benefited from including another time point, in Q4 of 2021, following the introduction of vaccines and the further relaxing of restrictions in Ireland. However, this was not possible due to time and resource constraints with this project. Future researchers should consider plotting the change in complaints in the years before and since the onset of the COVID-19 pandemic. The study is also limited in that it only examined complaints from one hospital. This did provide some consistency to the complaints and is useful for the hospital management itself; however, it does limit the generalisability of the study to other hospitals nationally and internationally. Despite this limitation, the hospital in question appeared to reflect national trends of reduced admissions in 2020 [29], and therefore, it is likely that some generalisations could be made. Further research should include data from other hospitals nationally and perhaps also internationally to extend the findings. More data could also allow for increased detail to be included in the published reports, as this study was limited in the detail it could provide due to the risk of making the small sample of incidents in question identifiable. Finally, the use of only one measure of safety (patient complaints) is a limitation of the study. It is clear that despite massive systemic changes to the workings of the hospital, patient complaints were only able to capture some of this change. It was also difficult to explain some of the findings of the complaint analysis in isolation. Combining a complaint analysis with other measures of safety such as staff incident reports or audits could help to contextualise the findings [17, 41] and provide a more holistic overview of the impact of COVID-19 on patient safety.

## Conclusion

The COVID-19 pandemic has had a huge impact on the delivery of healthcare in Ireland and internationally. However, despite the large changes in the delivery of healthcare, what patients complained about was largely unchanged. Although there was a decrease in the number of complaints per admission during the second wave of the pandemic as compared to before the pandemic, the greatest proportion of complaints remained concerned with institutional processes such as access to services and problems with bureaucracy—despite the dramatic changes in the healthcare system over this time. The severity of complaints was also significantly higher in Q4 2019 than in Q4 2020, which requires further exploration as the reasons for this difference are unclear. The health system, in seeking service user feedback, has a responsibility and obligation to act to resolve the system’s identified risks and improve patient experiences in the service. The findings of this study provoke further questions and considerations for research into the use of complaint data in the measurement and monitoring of safety and quality of care during emergencies such as the COVID-19 pandemic.
